# Supplementary material for: Gut microbiota DPP4-like enzymes are increased in type-2 diabetes and contribute to incretin inactivation
Source: Genome Biol. 2024 Jul 3;25:174. doi: 10.1186/s13059-024-03325-4 (PMC11221189; doi:10.1186/s13059-024-03325-4)
Supplement: Supplementary file 1 — Additional file 1: Figure S1. Comparison of DPP4-like function-associated peptidases. Figure S2. Parabacteroides merdae recombinant proteins and activity. Figure S3. Gliptin inhibition kinetics on Parabacteroides merdae DPP4-like protein. Table S1. Kinetics fold-change upon gliptin inhibition on pmDPP4 and hsDPP4 enzymes. [file 13059_2024_3325_MOESM1_ESM.pdf]

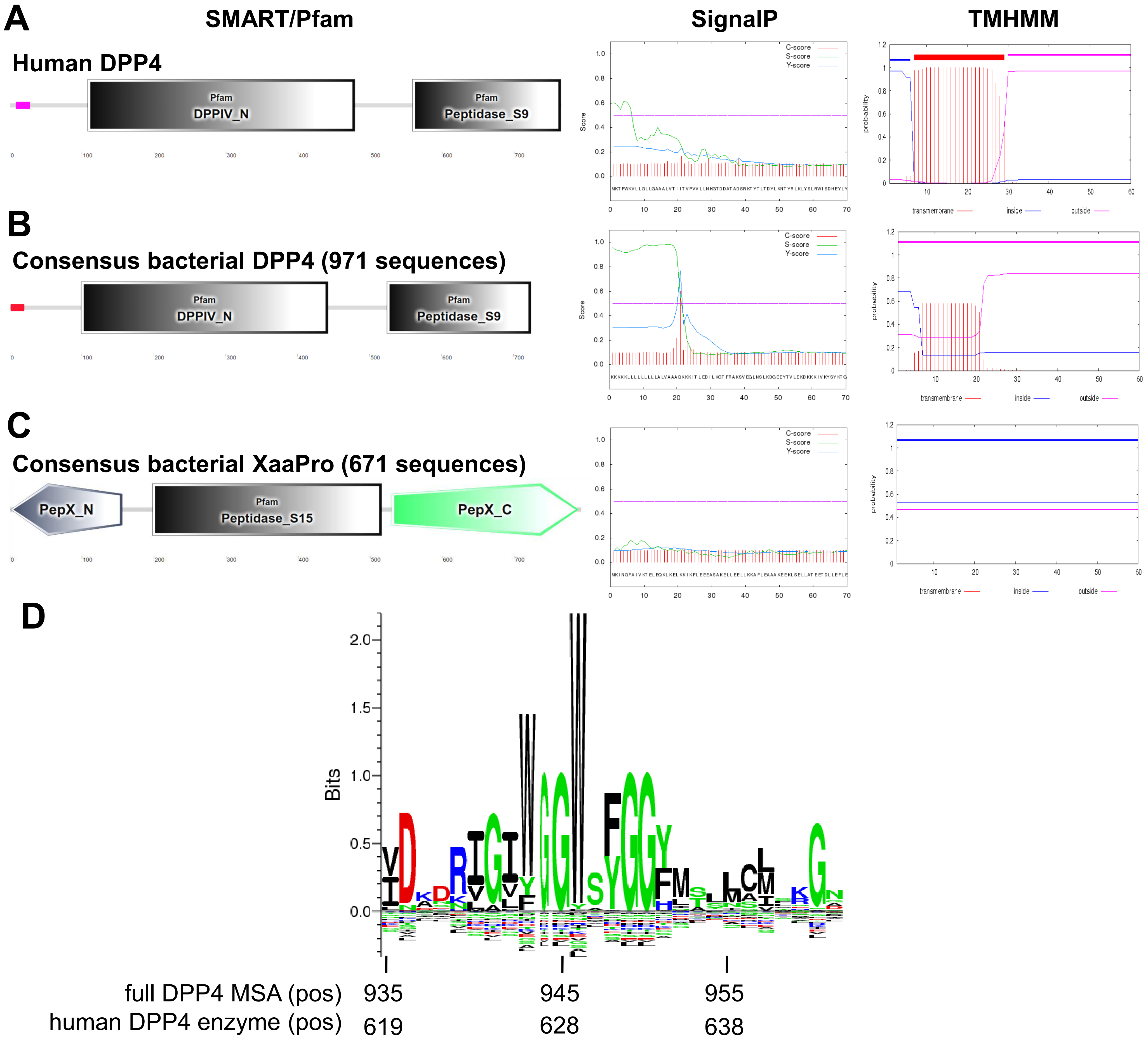

Figure S1. Comparison of DPP4-like function-associated peptidases. Domain architecture, signal peptide presence and transmembrane domain predictions were examined on the human DPP4 enzyme (SwissProt id P27487) (**A**), the consensus amino acid profile of bacterial DPP4-like proteins (**B**), and the consensus amino acid profile of PepX peptidases (**C**) using the respective web servers (top labels). Individual functional domains according to SMART and/or Pfam annotation present in full-length query proteins are presented in classical architecture diagrams. SignalP prediction for the N-terminal 70 amino acids shows C- (raw cleavage site score, red lines), S- (signal peptide score, green lines), and Y- (combined cleavage site score, blue lines) scores to determine the individual probability of amino acids to be part of a signal peptide driving protein secretion or potential membership of a transmembrane structure. TMHMM prediction for the N-terminal 70 amino acids shows the probability of individual amino acids to be part of alpha helices embedded in biological membranes, thus indicating localization in the transmembrane (red lines), inside of cell (blue line) or outside of cell (pink lines). The amino acid logo representation of the multiple sequence alignment (MSA) of DPP4-like proteins (bacteria, mouse, rat, and human) for the catalytic motif of S9 peptidase family is drawn in **D**. Amino acid logo was created using the positions between amino acids 935 and 941 of the MSA using the SeqLogo sever (http://www.cbs.dtu.dk/biotools/Seq2Logo/) and based on the P-Weighted Kullback-Leibler score. The amino acid positions for the human protein are also shown at the bottom. This regions shows the MEROPS [22] catalytic motif (GWSYGGY) of these serine proteases (S9).

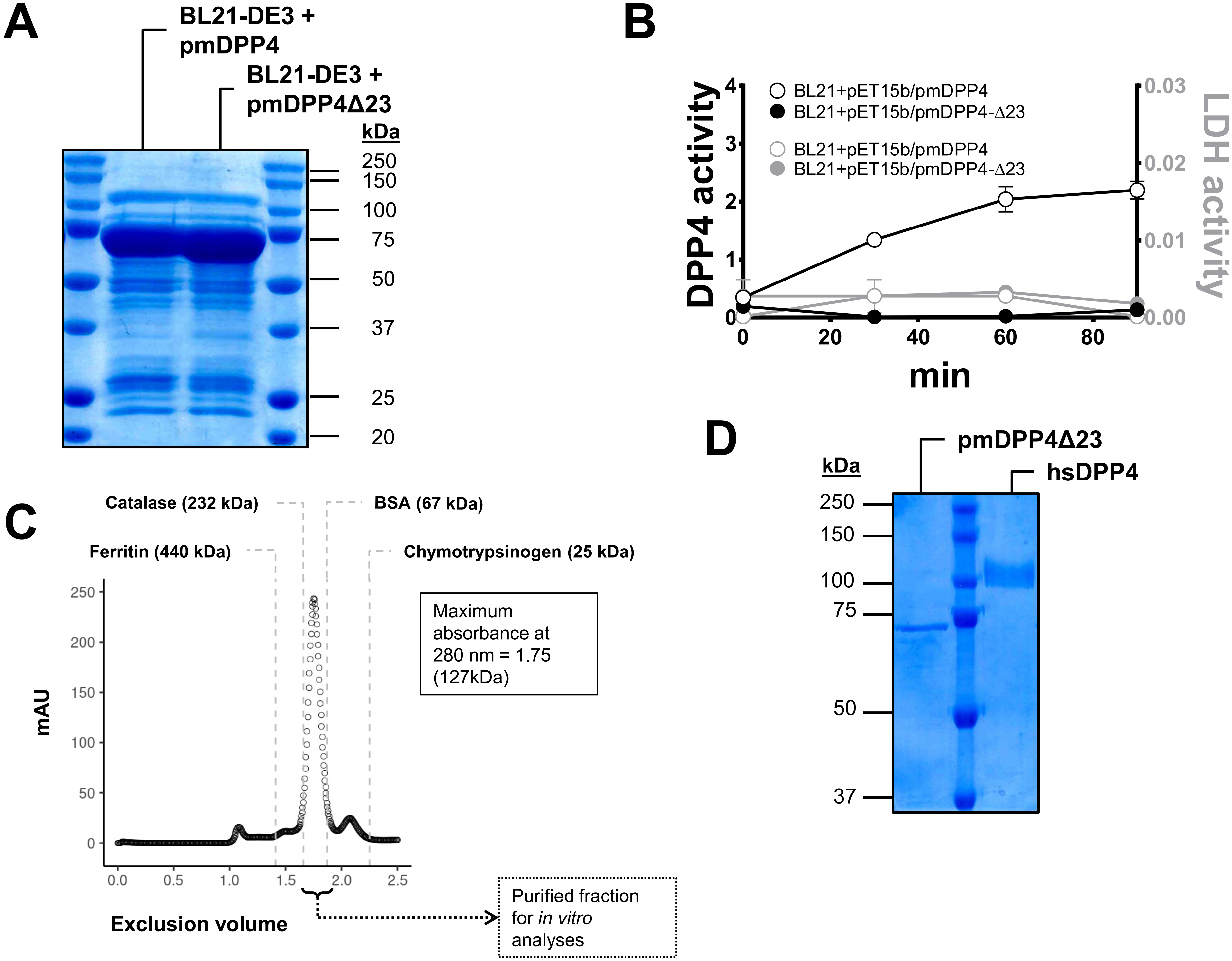

Figure S2. A, SDS-PAGE analysis of *E. coli* BL21-DE3 cell extracts expressing recombinant pmDPP4 and pmDPP4Δ23 proteins. **B**, Ninety minute kinetics of pmDPP4 activity (left y-axis, in terms of mU/mL units) and LDH activity (right y-axis, in terms of 490 nm - 680 nm absorbance) mediated by cell-free supernatants from *E. coli* BL21 cells carrying pET15b/pmDPP4 or pET15b/pmDPP4Δ23 constructs upon induction. **C**, Gel filtration assay of an ionic-exchange purified fraction of the pmDPP4Δ23 protein, revealing a dimeric nature in solution according to the calculated MW from the standards elution profile. **D**, Purified pmDPP4Δ23 and human DPP4 proteins used in the *in vitro* assays for human hormone hydrolysis and gliptin inhibition. Gels were stained with Coomassie blue to visualize protein bands. The Dual Xtra pre-stained protein standard (Bio-Rad, Hercules, CA) was used as a molecular size reference. LDH, lactate dehydrogenase.

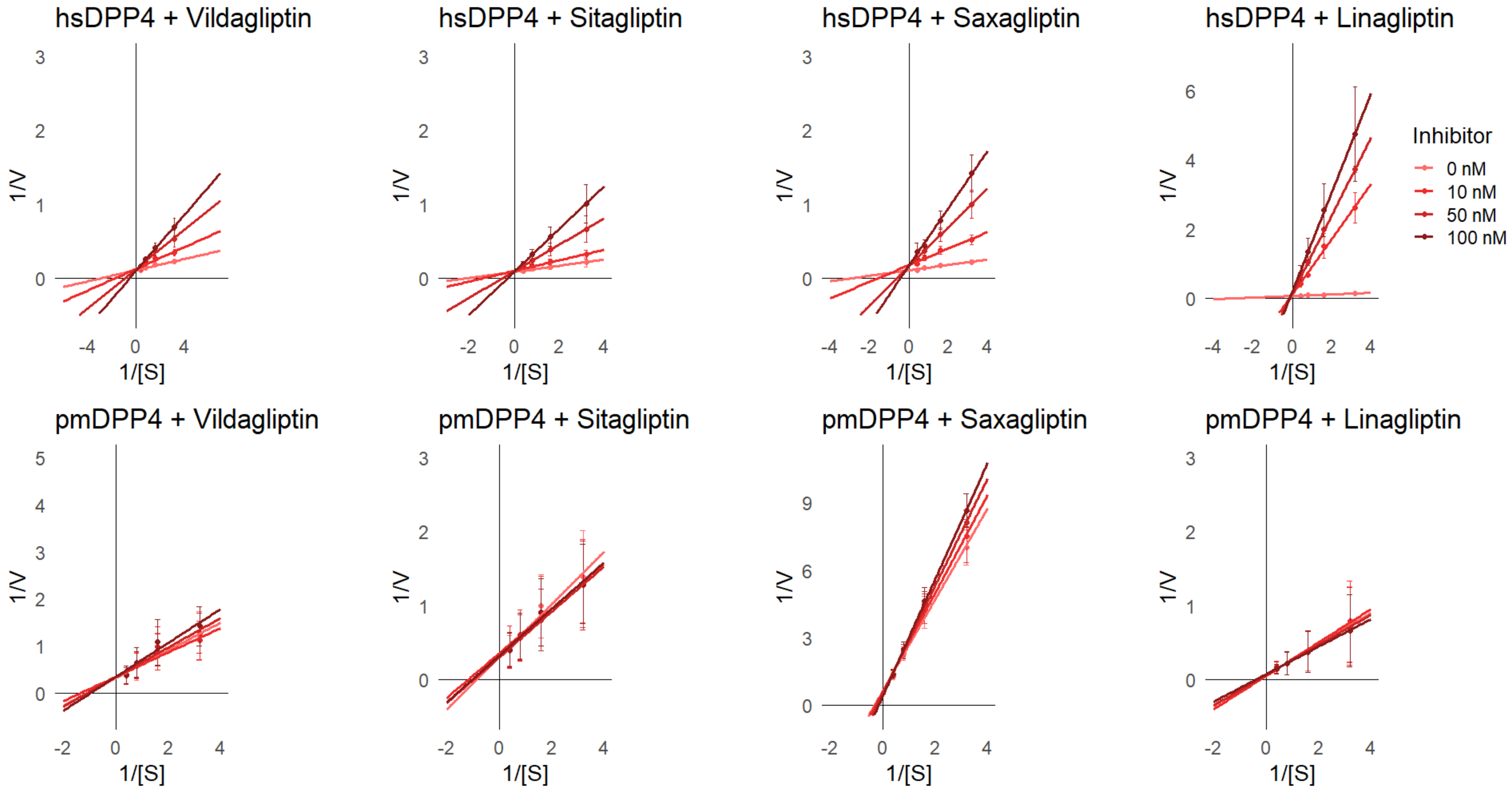

Figure S3. Gliptin inhibition kinetics on pmDPP4Δ23. Lineweaver-Burk double-reciprocal (1/V and 1/S) plots showing pmDPP4Δ23 (bottom panels) and hsDPP4 (top panels) gpPNA hydrolysis in presence of vildagliptin, sitagliptin, saxagliptin, and linagliptin. Inhibitor concentration is shown as line legend color from lower (0 nM, light red) to higher (100 nM, burgundy lines) concentrations. The apparent K_m was calculated respectively from inhibitor-associated data points using linear regression to find intersection point at the y-axis (1/S). V, reaction velocity (nmol PNA min^{-1} pmole enzyme^{-1}); S, substrate concentration (mM gpPNA).

Enzyme	Inhibitor (nM)	Km (mM)	SD	Fold-change	P-value (t.Test)
hsDPP4	Vildagliptin 100 nM	1.94	0.41	4.82	0.021
hsDPP4	Vildagliptin 50 nM	1.36	0.12	3.07	0.004
hsDPP4	Vildagliptin 10 nM	0.59	0.07	0.76	0.019
hsDPP4	Vildagliptin 0 nM	0.33	0.02	0.00	
hsDPP4	Sitagliptin 100 nM	4.35	0.60	12.58	0.007
hsDPP4	Sitagliptin 50 nM	2.15	0.27	5.73	0.007
hsDPP4	Sitagliptin 10 nM	0.72	0.03	1.24	0.000
hsDPP4	Sitagliptin 0 nM	0.32	0.03	0.00	
hsDPP4	Saxagliptin 100 nM	3.34	1.04	10.53	0.037
hsDPP4	Saxagliptin 50 nM	1.58	0.31	4.46	0.018
hsDPP4	Saxagliptin 10 nM	0.63	0.05	1.17	0.006
hsDPP4	Saxagliptin 0 nM	0.29	0.00	0.00	
hsDPP4	Linagliptin 100 nM	7.34	1.33	19.01	0.012
hsDPP4	Linagliptin 50 nM	8.24	1.10	21.48	0.006
hsDPP4	Linagliptin 10 nM	9.80	2.50	25.74	0.023
hsDPP4	Linagliptin 0 nM	0.37	0.05	0.00	
pmDPP4	Vildagliptin 100 nM	2.84	0.13	0.78	0.076
pmDPP4	Vildagliptin 50 nM	2.02	0.18	0.27	0.377
pmDPP4	Vildagliptin 10 nM	1.12	1.35	-0.30	0.595
pmDPP4	Vildagliptin 0 nM	1.60	0.65	0.00	
pmDPP4	Sitagliptin 100 nM	1.51	0.15	-0.15	0.301
pmDPP4	Sitagliptin 50 nM	1.44	0.16	-0.19	0.214
pmDPP4	Sitagliptin 10 nM	1.25	0.17	-0.30	0.096
pmDPP4	Sitagliptin 0 nM	1.79	0.34	0.00	
pmDPP4	Saxagliptin 100 nM	4.54	0.99	1.73	0.036
pmDPP4	Saxagliptin 50 nM	2.63	0.22	0.58	0.552
pmDPP4	Saxagliptin 10 nM	1.85	0.45	0.11	0.012
pmDPP4	Saxagliptin 0 nM	1.66	0.07	0.00	
pmDPP4	Linagliptin 100 nM	1.73	0.08	0.18	0.108
pmDPP4	Linagliptin 50 nM	1.66	0.11	0.13	0.196
pmDPP4	Linagliptin 10 nM	1.51	0.25	0.03	0.823
pmDPP4	Linagliptin 0 nM	1.46	0.18	0.00	

Table S1. K_m fold-change on pmDPP4Δ23 and hsDPP4 enzymes upon gliptin inhibition. Data derived from kinetics assays shown in Figure S3 are shown in this spreadsheet. Headers indicate enzyme source, inhibitor tested, K_m estimated, standard deviation (SD), fold-change, and p-value obtained from comparisons with respective reactions with no inhibitor.
